# Decreased ribosomal DNA transcription in dorsal raphe nucleus neurons differentiates between suicidal and non-suicidal death

**DOI:** 10.1007/s00406-015-0655-4

**Published:** 2015-11-21

**Authors:** Marta Krzyżanowska, Johann Steiner, Karol Karnecki, Michał Kaliszan, Ralf Brisch, Marek Wiergowski, Katharina Braun, Zbigniew Jankowski, Tomasz Gos

**Affiliations:** Department of Forensic Medicine, Medical University of Gdańsk, ul. Dębowa 23, 80-204 Gdańsk, Poland; Department of Psychiatry, Otto-von-Guericke-University, Magdeburg, Germany; Department of Zoology/Developmental Neurobiology, Institute of Biology, Otto-von-Guericke-University, Magdeburg, Germany

**Keywords:** Post-mortem, Suicide, Dorsal raphe nucleus, AgNOR staining

## Abstract

An involvement of the central serotonergic system has been implicated in the pathogenesis of suicide. The dorsal raphe nucleus (DRN) is the main source of serotonergic innervation of forebrain limbic structures disturbed in suicidal behaviour. The study was carried out on paraffin-embedded brainstem blocks containing the DRN obtained from 27 suicide completers (predominantly violent) with unknown psychiatric diagnosis and 30 non-suicidal controls. The transcriptional activity of ribosomal DNA (rDNA) in DRN neurons as a surrogate marker of protein biosynthesis was evaluated by the AgNOR silver staining method. Significant decreases in AgNOR parameters suggestive of attenuated rDNA activity were found in the cumulative analysis of all DRN subnuclei in suicide victims versus controls (*U* test *P* values < 0.00001). Our findings suggest that the decreased activity of rDNA transcription in DRN neurons plays an important role in suicide pathogenesis. The method accuracy represented by the area under receiver operating characteristic curve (>80 %) suggests a diagnostic value of the observed effect. However, the possible application of the method in forensic differentiation diagnostics between suicidal and non-suicidal death needs further research.

**Electronic supplementary material:**

The online version of this article (doi:10.1007/s00406-015-0655-4) contains supplementary material, which is available to authorized users.

## Introduction

Disturbances of the central serotonergic system are implicated in a multifaceted way in suicidal behaviour (for reviews see: [1, 2]), which has been proposed to be an independent mental disorder in the fifth edition of the Diagnostic and Statistical Manual of Mental Disorders—DSM V [3] in accordance with numerous neurobiological research data (for reviews see: [1, 2]).

As revealed by neuropathological research on suicide, abnormalities in the serotonergic system may be structurally restricted to a specific brain region, the dorsal raphe nucleus (DRN), which affects brain circuits (for a review see: [4]). DRN neurons provide the major serotonergic innervation to the prefrontal cortex (PFC) [5–7], which plays a pivotal role in behavioural regulation. Limbic regions of the PFC (i.e. the anterior cingulate cortex and the orbitofrontal cortex) in turn may reciprocally regulate DRN function through direct pyramidal input [8, 9] modulated by serotonergic receptors in the PFC (for reviews see: [10–13]).

A number of post-mortem studies revealed changes in the DRN of suicide victims [14–28]. However, discrepancies resulting from various factors exist in the studies. Inconsistencies regarding psychiatric diagnosis, treatment, cause of death, the delineation of investigated areas, methodical issues and small sample sizes seem to be the most significant [29].

Nucleolar organising regions (NORs) are genetic loci on chromosomes that are composed of ribosomal DNA (rDNA) and proteins, some of which are argyrophilic. In human interphase cells, silver-stained NORs (AgNORs) clustered together in the nucleolus represent the site of transcriptionally active NORs and ribosomal RNA synthesis, which constitutes approximately one half of entire transcriptional activity in the cell. In the AgNOR staining evaluated by light microscopy, AgNORs are indistinguishable from each other and form the AgNOR area. They are located in the nucleolar area, but smaller than this area (compared, for instance, with haematoxylin–eosin and Nissl staining [30]). As a surrogate marker of protein biosynthesis and an important sensor of cellular stress of different nature, the transcriptional activity of rDNA can be assessed by measuring AgNOR parameters. These are: AgNOR area (representing the nucleolus), AgNOR number (i.e. the number of AgNOR areas within one nucleus) and AgNOR ratio defined as the quotient of total AgNOR area in the nucleus and nuclear area [18, 24, 31–42] (for reviews see: [43, 44]).

A key role of rDNA transcriptional activity in neuronal plasticity has been proven in neuronal culture [45], and molecular studies have revealed that this activity is decreased in the suicidal hippocampus [46]. Our previous AgNOR studies of the DRN [24, 47] and other brain structures [33–37] in depression have suggested disturbed (predominantly decreased) rDNA transcription in neurons, specifically in suicidal patients, which is consistent with molecular results [46].

In the present study, we hypothesised a decreased rDNA transcriptional activity in DRN neurons in suicide completers regardless of their underlying psychiatric diagnosis (i.e. independent on psychiatric comorbidity) and tested this hypothesis by the application of the AgNOR staining method in our forensic post-mortem material. We aimed at both basic research on the neurobiology of suicide and the evaluation of possible diagnostic usefulness of the method in the differentiation between suicidal and non-suicidal death.

## Materials and methods

### Human brain tissue

Brainstems of suicide victims with unknown psychiatric comorbidity (typical for most of the suicide cases autopsied in our Department of Forensic Medicine) and sudden death controls were obtained during routine forensic autopsies in accordance with existing EU law regulations. The study has been approved by the local ethics committee of the Medical University of Gdańsk and performed in accordance with the ethical standards laid down in the Declaration of Helsinki of 1989.

Detailed diagnostic, demographic and toxicological data together with raw data of AgNOR measurements are presented in Supplementary Table. Violent suicide methods prevailed in the suicide cohort (23 out of 27), which are representative for our autopsy material. All brains were free of gross neuropathology suggestive of vascular, traumatic, inflammatory, neoplastic and neurodegenerative processes. A toxicology screening on blood and urine for ethanol was performed at each autopsy. The majority of investigated cases (17 suicide victims and 28 controls, see Supplementary Table) revealed the blood alcohol concentration (BAC) below the limit of quantification (LOQ), i.e. <0.2 g/l according to internationally accepted analytical guidelines. Other substances of abuse, antidepressant and antipsychotic drugs, as well as their metabolites were investigated when an intoxication was suggested by the scene inspection and/or other available information sources prior to the autopsy, i.e. in three cases. These cases together with a helium inhalation victim constituted the non-violent suicide subgroup (see Supplementary Table).

Brainstems were isolated from the brains and fixed in toto in 10 % phosphate-buffered formaldehyde for 1 week. After being fixated, tissue blocks containing the entire DRN were isolated from the brainstems and embedded in paraffin. Subsequently, serial 5-µm-thick transverse sections were cut along the entire rostrocaudal axis of the DRN. Every 200th section was mounted and stained for AgNOR.

### AgNOR staining

**Fig. 1:**
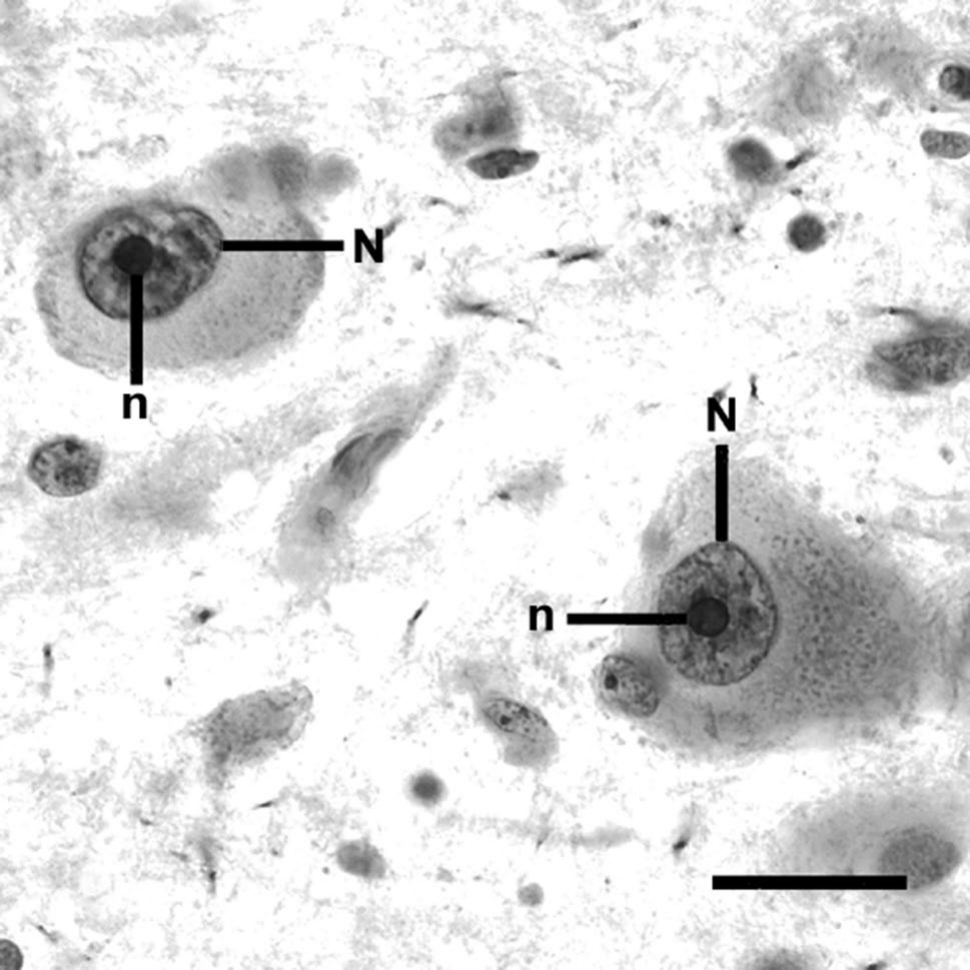
After AgNOR staining, the borders of AgNOR areas (representing nucleoli) (n) are clearly visible within nuclei (N) of DRN neurons (control case, interfascicular subnucleus, *scale bar* 20 µm). The differences in AgNOR parameters were beyond qualitative evaluation, and they could only be captured by means of quantitative measurements

Silver staining was carried out as previously described [24]. Briefly, 5-µm paraffin sections were dewaxed and rehydrated through graded alcohols. The silver staining was freshly prepared by dissolving 2 g/dl gelatin in 1 ml/dl aqueous silver nitrate solution at a 1:2 ratio. The sections were incubated with this solution in a dark moist chamber at room temperature for 45 min and subsequently washed with deionised water. Following this protocol, the AgNOR area—containing AgNORs (that are clustered, undistinguishable from each other) and representing the nucleolus—appears as an intranuclear, clearly delineated black or dark brown small spot, and the nuclear border is clearly visible in the majority of large DRN neurons (Fig. 1), which are most probably serotonergic output neurons [48]. Glia cells were distinguished from neurons according to the established cytomorphological criteria [49].

### Quantification

In each of the DRN subnuclei (ventral, ventrolateral, dorsal, interfascicular and caudal [48]), AgNOR parameters were determined in 40 neurons with clearly visible borders of the nucleus and AgNOR areas (Fig. 1), which were selected throughout the available AgNOR-stained sections, i.e. in seven sections for a case, on average. Thus, the AgNOR parameters of 200 DRN neurons were investigated in each case. The number of investigated neurons was established arbitrarily in accordance with the guidelines on quantitative evaluation and diagnostic and research studies employing the AgNOR method. This method does not require an estimation of the number of cells and/or nuclei [24]. The neurons were sampled using a 400× magnification. The AgNOR areas (composed of clustered AgNORs and representing the nucleoli), their number and the nuclear area within a single-sampled neuron were determined using a light microscope attached to a computer image analysis system (cellSens^®^, Olympus, Japan). In this system, each of the neurons sampled by 400× magnification was visualised digitally and focused, and the sharpest and longest profiles of the nucleus and AgNOR areas were traced by the mouse cursor on the screen. As a result, the numerical values of AgNOR and the nuclear areas and the numbers of AgNOR areas were calculated automatically. Subsequently, the AgNOR ratio (relative AgNOR area) was derived by dividing the total AgNOR area by the nuclear area, taking into account all the AgNOR areas present per neuronal nucleus. This procedure was performed separately for each of the sampled neurons. The sampled measures were averaged to derive a single set of values for each DRN subnucleus, for the rostral and caudal subdivisions of the DRN and for the entire DRN as a single anatomical structure in each of the investigated cases.

### Data analysis

Statistical analyses were performed with the data analysis software system STATISTICA version 10 (StatSoft^®^, Inc. 2011, www.statsoft.com). As normal distribution was not given for all analysed AgNOR parameters, nonparametric statistical procedures were used. Unadjusted two-way comparisons with the Mann–Whitney *U* test were carried out to detect between-group differences.

The *χ*^*2*^ test and the *U* test were used to detect the possible differences between the study groups with respect to sex, age, season of the year (month of death in spring/summer vs. autumn/winter), brain weight, post-mortem delay and BAC (whose values below LOQ were accounted null values in statistical analysis). All statistical tests were two tailed.

Spearman correlation coefficients were calculated to determine the impact of *numerical variables* which might confound the dependent variables.

Generally, *P* values of <0.05 were accepted as statistically significant.

STATISTICA Automated Neuronal Networks (SANN) module containing receiver operating characteristic (ROC) procedure was applied for the evaluation of potential diagnostic value of the method, i.e. its accuracy (represented by the area under ROC curve), sensitivity and specificity.

## Results

### Qualitative analysis of the AgNOR staining

After AgNOR staining of the DRN neurons, borders of the AgNOR area (containing AgNORs that were clustered together and indistinguishable from each other) were clearly visible (Fig. 1) in line with the staining patterns in DRN neurons presented previously [24]. Most of the neurons contained one AgNOR area (representing the nucleolus in this staining method). Two or more AgNOR areas were observed very rarely, which explains why the AgNOR number was near 1.

### Quantitative analysis of the AgNOR staining

The differences in AgNOR parameters were beyond qualitative evaluation, and they could only be captured by means of quantitative measurements.

The statistical analysis revealed highly significant differences by means of the cumulative analysis of all DRN subnuclei. Both the AgNOR area and the relative AgNOR area were decreased in suicide victims compared to controls (Table 1). No significant differences existed in any of the AgNOR parameters between violent (*n* = 23) and non-violent (*n* = 4) suicide victims. As revealed by the ROC curve for the AgNOR area, the method accuracy (represented by the area under ROC curve, AUC) is 83 %; its sensitivity according to the suicide diagnosis is 67 %, whereas the specificity of suicide exclusion (i.e. the diagnosis of non-suicidal case) is 90 %. The respective values for the relative AgNOR area are: 85 % (AUC), 78 % (sensitivity) and 90 % (specificity), and for both parameters when tested simultaneously they are: 89 % (AUC), 85 % (sensitivity) and 87 % (specificity).

**Table 1:** Presentation of significant diagnostic group differences regarding the evaluation of rDNA transcriptional activity in dorsal raphe nucleus neurons by the AgNOR staining (for the detailed values of parameters see Supplementary Table 1)

Parameter and group	Parameter values	*U* test *P*
	Median (*q*1, *q*3, *n*)	S/C
Nuclear area in µm^2^		ns
S	117.425 (105.715, 127.415, 27)	
C	113.619 (101.426, 127.506, 30)	
AgNOR area in µm^2^ per nucleus		0.000007
S	10.201 (9.349, 11.552, 27)	
C	12.315 (11.443, 13.178, 30)	
Number of AgNORs per nucleus		ns
S	1.000 (1.000, 1.019, 27)	
C	1.000 (1.000, 1.012, 30)	
Relative AgNOR area		0.000002
S	0.086 (0.081, 0.092, 27)	
C	0.102 (0.099, 0.113, 30)	

*S* suicide victims, *C* controls, *q1* and *q3* quartile 1 and 3, *n* number of cases, *U* test *P* *U* test *P* values, *ns* non-significant

### Confounders

Variables which could influence the AgNOR parameters in the DRN neurons, such as post-mortem interval (PMI), sex, age at death, season of the year (month of death in spring/summer vs. autumn/winter) and brain weight were not associated with the values of the AgNOR parameters in the compared groups. No significant differences in these variables between suicides and controls were revealed. As the PMI is a very important confounder in post-mortem research, the hypothetical influence of this factor was excluded by an additional analysis of data. The cases with longest PMI (i.e. ≥70 h, 6 cases in suicidal and 3 in control group, see Supplementary Table) were separated, and AgNOR parameters were compared between them and remaining cases in respective groups, which revealed non-significant *U* test *P* values. Moreover, intergroup comparisons were performed after exclusion of these cases, which supported the highly significant differences between suicidal and control groups in both the AgNOR area (*U* test *P* value 0.000046) and the relative AgNOR area (*U* test *P* value 0.000007; for a comparison see Table 2).

**Table 2:** Summarised data on the confounding variables analysis between suicide victims (*n* = 27) and control subjects (*n* = 30)

	Sex	Age (year)	PMI (h)	BAC (g/l)
Intergroup comparisons				
Suicide victims: ratio/median (*q*, *q*3)	23 m/4f	43 (28, 53)	24 (24, 48)	0.0 (0.0, 2.1)
Controls: ratio/median (*q*1, *q*3)	11 m/19f	48 (39, 61)	24 (24, 48)	0.0 (0.0, 0.0)
Statistics (suicide victims *versus* Controls)				
Test	*χ* ^2^ test	*U*	*U*	*U*
Characteristic value	*χ* ^2^ = 13.90	*Z* = 1.886	*Z* = − 0.280	*Z* = 1.846
*P* value	0.0002	0.059	0.780	0.065

AgNOR parameters	Group		Age	PMI	BAC
Correlation analysis between numerical confounding variables listed above and AgNOR parameters revealing significant intergroup differences					
AgNOR area	S	*r/P*	0.16/0.42	−0.05/0.80	0.06/0.77
	C	*r/P*	0.11/0.57	−0.20/0.28	0.18/0.33
Relative AgNOR area	S	*r/P*	−0.14/0.47	0.08/0.70	0.28/0.16
	C	*r/P*	0.09/0.62	−0.04/0.85	−0.01/0.97

*f* female, *m* male, *q1* and *q3* quartile 1 and 3, *PMI* post-mortem interval, *BAC* blood alcohol concentration, *S* suicide victims, *C* controls, *r* correlation coefficient and *P P* value of the Spearman’s correlation, *PMI* post-mortem interval, *BAC* blood alcohol concentration

Although males unequivocally prevailed in suicidal versus control group (*χ*^2^ test *P* value 0.0002, Table 2), no intragroup differences related to sex were found in any of AgNOR parameters, among them the AgNOR area and the relative AgNOR area (non-significant *U* test *P* values).

## Discussion

Our study revealed a significantly decreased AgNOR area in DRN neurons suggestive of their decreased rDNA transcriptional activity in suicide victims versus controls. The observed effect was not confounded by other variables, among them post-mortem interval. The significance was shown in the cumulative analysis of all DRN subnuclei. This phenomenon could be related to the characteristic of DRN connections with target structures which overlap [5–7] in spite of the accentuated distinctiveness [50, 51].

The results are in line with our previously published findings, which suggested a decreased rDNA transcription in DRN neurons specific to depressed suicide completers (from both major depressive disorder and bipolar disorder diagnostic groups of affective disorders) compared to non-suicidal depressed patients from these diagnostic groups [24]. Our recent findings in schizophrenia suggest a similar effect in suicidal compared to non-suicidal patients [52]. Therefore, our both previous and present results may suggest that the decreased rDNA transcription in DRN neurons is accentuated in suicide regardless of psychiatric comorbidity. In this way, they correspond also with the well-established view on serotonergic hypofunction as a diagnose-overreaching neurobiological phenomenon symptomatic of suicidal behaviour [1, 2, 4].

Regardless of the high statistical significance of our results (*U* test *P* < 0.00001), both the AgNOR area and the AgNOR ratio values overlapped between the suicide and non-suicide cohorts. Nevertheless, the area under ROC curve higher than 80 % with similar sensitivity and specificity values suggests a diagnostic value of present AgNOR technique [53]. However, the confirmation of diagnostic relevance of the method needs further study of more numerous cohorts.

The rDNA transcription corresponds with the growth of both neuronal processes and neuronal body [45]. Therefore, our results are concurrent with those previous neuropathological studies, where a decrease in serotonergic axonal endings in the PFC [54–56] and the diminished neuronal body size in the DRN [26] were found in suicide victims.

On the other hand, the undisturbed or augmented expression of the key enzyme in serotonin synthesis, tryptophan hydroxylase (TPH), in DRN neurons in suicide was reported [16–18, 20, 22, 23, 28] (for a review see: [4]), which corresponds with the increased serotonin level found in DRN homogenates in suicide victims [57]. The production of neurotoxic metabolites subsequent to the increased intracellular serotonin concentration may damage the DNA of DRN neurons [58], which constitutes a potent inhibitor of rDNA transcription [59]. Therefore, one consequence of the increased TPH and subsequently augmented serotonin level in DRN neurons may be their decreased rRNA synthesis followed by deteriorated arborisation of serotonergic axons [60] in PFC regions.

The interpretation of our current results is not unequivocal due to the complicated nature of DRN activity regulation. The observed effect may be related to disturbances in 5HT2A receptor (5HT2AR) in limbic PFC areas, which provide the most afferents to the DRN [8, 61]. The up-regulation of 5HT2ARs in the limbic PFC was observed in suicide [62] (for reviews see: [29, 63, 64]). In experimental models, the hyperfunction of these receptors on pyramidal neurons provides their increased output with subsequent inhibition of DRN neurons [65, 66], which may result in the attenuated rDNA transcription observed in our suicidal cohort.

On the other hand, experimental research on animal models suggests a sophisticated interaction between distal afferents and DRN neurons with an involvement of local circuits [65–71]. These local circuits may be dysregulated in suicide and thus contribute to the observed effect. The increased expression of presynaptic 5-HT1A auto-inhibitory receptors in the DRN in suicide may constitute one of the important local factors [72, 73]. The regionally disturbed function of corticotropin-releasing hormone (CRH) could also contribute to the decreased rDNA transcription in DRN neurons in suicide [15] (for reviews on CRH function in the DRN see: [70, 74]).

The above-referenced findings point to a partially diminished protein synthesis as a consequence of the suggested rDNA activity decrease in the DRN in suicide. However, in a view of cited studies, the deteriorated rDNA activity in DRN neurons might play a fundamental role in the decrease of serotonergic terminals in the PFC. This abnormality provides a morphological substrate for the disturbed serotonergic neurotransmission in the PFC with profound consequences for the behavioural regulation. Therefore, the decreased rDNA activity with subsequently diminished plasticity of DRN neurons may constitute one of the key factors in the serotonergic dysfunction in suicide.

### Limitations

The present study has certain limitations that have to be considered: (1) A relatively small number of cases have been analysed, especially for the evaluation of potential diagnostic value of the method; therefore, results have to be confirmed in a larger sample. (2) The psychiatric diagnoses (also according to substance use disorders) and the data on possible psychotropic medication preceding death were not available. The levels of psychotropic drugs were established only in three suicide victims where medication overdose constituted a cause of death. However, our current study did not aim at the relation between suicide and other mental disorders, and our previous studies did not suggest that the decreased AgNOR area in DRN neurons may be related to the medication in the last three months of life [24]. (3) The application of paraffin-embedded tissue is a limitation of our method compared to frozen brain samples, which would allow the application of a wider set of approaches. (4) Although serotonergic neurons comprise about 70 % of DRN neurons [7] and nearly all the large cells in this nucleus are serotonergic [5, 48], we could not determine the type of selected neurons by AgNOR staining. Since AgNOR areas correspond almost 100 % to the nucleoli in DRN neurons, immunohistochemistry for tryptophan hydroxylase with a Nissl counterstain could bring to light, whether the cells with reduced AgNOR areas are really serotonergic. Furthermore, confocal immunofluorescence microscopy using antibodies against nucleolin and tryptophan hydroxylase could be another approach to resolve this issue.

## Conclusion

In summary, our results suggest decreased rDNA transcription in DRN neurons in suicide as a presumable consequence of disturbed inputs to the DRN and/or their local transformation. The present method could probably aid forensic differentiation diagnostics between suicidal and non-suicidal death in cases where traditional methods do not solve this problem. However, the further research is warranted to appropriately evaluate this issue.

## Electronic supplementary material

Below is the link to the electronic supplementary material.

Supplementary material 1 (PDF 165 kb)
