# Supplementary material for: Decreased ribosomal DNA transcription in dorsal raphe nucleus neurons differentiates between suicidal and non-suicidal death
Source: Eur Arch Psychiatry Clin Neurosci. 2015 Nov 21;266:217–24. doi: 10.1007/s00406-015-0655-4 (PMC4819736; doi:10.1007/s00406-015-0655-4)
Supplement: Supplementary file 1 — Supplementary material 1 (PDF 165 kb) [file 406_2015_655_MOESM1_ESM.pdf]

Decreased ribosomal DNA transcription in dorsal raphe nucleus neurons differentiates between suicidal and non-suicidal death

Marta Krzyżanowska, Johann Steiner, Karol Karnecki, Michał Kaliszan, Ralf Brisch, Marek Wiergowski, Katharina Braun, Zbigniew Jankowski, Tomasz Gos

European Archives of Psychiatry and Clinical Neuroscience

Corresponding author:

Tomasz Gos, MD

Department of Forensic Medicine

Medical University of Gdańsk

ul. Dębowa 23

80-204 Gdańsk, Poland

E-mail: gost@gumed.edu.pl

Supplementary Table Detailed diagnostic and demographic data and the values of parameters revealed by AgNOR staining in dorsal raphe nucleus neurons in suicide victims (n=27) and control subjects (n=30).
Abbreviations: f – female; m – male; q1 and q3 – quartile 1 and 3; PMI – postmortem interval; BAC – blood alcohol concentration; LOQ – limit of quantification (BAC = 0.2 g/l).

Case ID	Cause of death	Sex	Age [yr]	PMI [h]	BAC [g/l]	Nuclear area (μm^2)	AgNOR area (μm^2)	AgNOR number	Relative AgNOR area
	SUICIDE VICTIMS								
1	Hanging	m	47	24	3.3	120.034	9.261	1.000	0.078
2	Hanging	m	30	24	<LOQ	125.198	10.201	1.028	0.082
3	Hanging	m	45	24	0.9	101.807	9.367	1.000	0.091
4	Hanging	m	48	48	<LOQ	105.120	8.501	1.033	0.080
5	Hanging	m	43	24	2.1	89.652	6.765	1.020	0.076

6	Hanging	f	46	24	3.4	117.425	11.942	1.022	0.101
7	Hanging	m	57	24	<LOQ	134.127	11.669	1.042	0.089
8	Hanging	m	54	24	<LOQ	127.415	10.468	1.000	0.083
9	Hanging	m	23	24	<LOQ	104.996	9.135	1.007	0.089
10	Hanging	m	55	70	1.3	113.885	9.524	1.011	0.085
11	Hanging	m	23	24	1.0	103.747	9.350	1.000	0.092
12	Hanging	f	55	24	<LOQ	123.251	10.534	1.000	0.086
13	Hanging	m	27	72	<LOQ	132.076	11.552	1.019	0.089
14	Hanging	m	43	24	3.3	141.411	12.332	1.000	0.086
15	Hanging	m	44	48	<LOQ	123.324	10.335	1.000	0.086
16	Hanging	m	29	120	<LOQ	99.515	9.790	1.000	0.101
17	Hanging	m	27	96	2.2	97.269	9.648	1.000	0.100
18	Hanging	m	27	48	2.5	113.196	11.522	1.000	0.103
19	Fall from building	m	28	41	<LOQ	160.715	13.131	1.066	0.082
20	Fall from building	f	27	33	<LOQ	148.822	11.049	1.000	0.076
21	Self-harm by sharp object (cut wound of neck)	m	77	48	<LOQ	134.041	10.411	1.000	0.079
22	Self-harm by sharp object (multiple cut wounds of forearms)	m	61	96	<LOQ	119.967	9.975	1.000	0.086
23	Self-harm by handgun discharge (gunshot wound of thigh)	m	50	24	2.13	126.784	12.864	1.000	0.102
24	Self-poisoning by Midazolam	m	36	24	<LOQ	106.042	8.488	1.075	0.080
25	Self-poisoning by Amitriptyline	f	39	72	<LOQ	116.020	10.068	1.000	0.090
26	Self-poisoning by Carbamazepine	m	53	2	<LOQ	114.041	11.674	1.000	0.103

27	Self-poisoning by helium inhalation	m	29	24	<LOQ	109.008	8.685	1.014	0.081
Suicide victims: ratio/median (q1, q3)		*23m/4f*	*43 (28, 53)*	*24 (24, 48)*	*0.0 (0.0, 2.1)*	*117.425 (105.715, 127.415)*	*10.201(9.349, 11.552)*	*1.000 (1.000, 1.019)*	*0.086 (0.081, 0.092)*
	CONTROLS								
28	Coronary failure	m	47	24	<LOQ	96.067	14.572	1.012	0.137
29	Coronary failure	m	63	48	<LOQ	114.122	12.370	1.000	0.102
30	Coronary failure	f	61	8	<LOQ	145.418	14.784	0.998	0.101
31	Coronary failure	f	61	24	<LOQ	126.208	10.121	1.000	0.081
32	Coronary failure	f	65	24	<LOQ	144.607	12.114	1.000	0.084
33	Coronary failure	f	61	24	<LOQ	127.506	13.631	1.000	0.101
34	Acute myocardial infarction	m	47	24	<LOQ	95.494	11.443	1.000	0.117
35	Acute myocardial infarction	m	40	96	<LOQ	95.971	9.690	1.018	0.098
36	Acute myocardial infarction	f	64	26	<LOQ	108.399	11.516	1.000	0.101
37	Acute myocardial infarction	f	63	24	<LOQ	99.037	12.429	1.000	0.120
38	Acute myocardial infarction	f	39	48	<LOQ	135.098	14.248	1.067	0.103
39	Pulmonary embolism (acute cor pulmonale)	f	48	26	<LOQ	100.086	10.803	1.000	0.103
40	Pulmonary embolism (acute cor pulmonale)	f	30	48	<LOQ	117.920	12.386	0.997	0.098
41	Pulmonary embolism (acute cor pulmonale)	m	56	24	<LOQ	102.774	10.619	1.000	0.100
42	Pulmonary embolism (acute cor pulmonale)	m	54	24	<LOQ	145.587	11.683	1.078	0.086
43	Pulmonary embolism (acute cor pulmonale)	f	48	48	<LOQ	83.807	9.513	1.000	0.108
44	Sudden cardiac death	f	33	72	<LOQ	110.280	11.101	1.000	0.100
45	Sudden cardiac death	f	38	24	<LOQ	113.116	12.182	1.022	0.102

46	Sudden cardiac death	f	67	24	<LOQ	101.426	13.049	1.000	0.119
47	Sudden cardiac death	f	39	24	<LOQ	107.360	11.160	1.039	0.104
48	Sudden cardiac death	f	66	24	<LOQ	118.046	13.830	1.006	0.114
49	Ruptured abdominal aortic aneurysm	m	56	48	<LOQ	92.627	12.336	1.026	0.128
50	Ruptured abdominal aortic aneurysm	m	64	35	<LOQ	138.556	13.846	1.000	0.094
51	Ruptured abdominal aortic aneurysm	f	50	72	<LOQ	101.756	12.072	1.000	0.115
52	Stabbed (open wounds of thorax)	m	47	48	<LOQ	130.319	13.178	1.000	0.101
53	Stabbed (open wound of thorax)	m	28	7	4.12	111.798	12.396	1.013	0.111
54	Accidental fall from building	f	24	48	2.93	137.625	13.563	1.000	0.098
55	Injury of cervical spinal cord	f	19	20	<LOQ	126.908	12.294	1.000	0.097
56	Choked on food	m	23	48	<LOQ	125.267	12.632	1.000	0.103
57	Subarachnoid haemorrhage	f	39	48	<LOQ	116.472	11.511	1.000	0.099
Controls: ratio/median (q1, q3)		*11m/19f*	*48 (39, 61)*	*24 (24, 48)*	*0.0 (0.0, 0.0)*	*113.619 (101.426, 127.506)*	*12.315 (11.443, 13.178)*	*1.000 (1.000, 1.012)*	*0.102 (0.099, 0.113)*
Statistics	test	χ^2-test	U	U	U	U	U	U	U
	characteristic value	χ^2= 13.90	Z = 1.886	Z = −0.280	Z = 1.846	Z = 0.679	Z = −4.275	Z = 1.103	Z= −4.515
	P value	***0.0002***	*0.059*	*0.780*	*0.065*	*0.499*	***0.000007***	*0.336*	***0.000002***
